# Assessing the Relationships between Physical Activity, a Healthy Life, and Personal Happiness in European Union Countries

**DOI:** 10.3390/healthcare12191941

**Published:** 2024-09-27

**Authors:** Gabriel Ioan Mangra, Mădălina Giorgiana Mangra, Claudiu George Bocean, Anca Antoaneta Vărzaru

**Affiliations:** 1 Department of Theory and Methodology of Motor Activities, Faculty of Physical Education and Sport, University of Craiova, 200585 Craiova, Romania; gabriel.mangra@edu.ucv.ro; 2 Department of Finance, Banking and Economic Analysis, Faculty of Economics and Business Administration, University of Craiova, 200585 Craiova, Romania; madalina.mangra@edu.ucv.ro; 3 Department of Management, Marketing and Business Administration, Faculty of Economics and Business Administration, University of Craiova, 13 AI Cuza Street, 200585 Craiova, Romania; 4 Department of Economics, Accounting and International Business, Faculty of Economics and Business Administration, University of Craiova, 13 AI Cuza Street, 200585 Craiova, Romania

**Keywords:** physical activity, physical inactivity, healthy life years, life expectancy, personal happiness, artificial neural network analysis, cluster analysis

## Abstract

**Background:** Maintaining a physically active lifestyle is a determinant factor of a healthy life and personal happiness. Meanwhile, physical inactivity remains a significant issue, resulting in negative consequences for public health. **Objectives:** This paper investigates the relationships between physical activity, physical inactivity, a healthy life, life expectancy, and personal happiness in European Union (EU) countries. **Methods:** This empirical study uses an artificial neural network and cluster analysis to analyze and interpret data from 27 EU countries. Artificial neural network analysis enables the assessment of the relationships between physical activity and inactivity, a healthy life, and personal happiness, while cluster analysis identifies groups of EU countries based on physical activity, healthy life, and personal happiness indicators. **Results:** The results show significant positive links between physical activity and improvements in healthy living and personal happiness. **Conclusions:** This study highlights considerable variations among EU countries regarding the levels of physical activity, healthy living, and personal happiness, emphasizing the importance of promoting physical activity to enhance public health and overall well-being. The findings suggest the need to develop customized policies that address country-specific factors and promote an active lifestyle.

## 1. Introduction

The significance of physical activity (PA) for health and well-being has emerged as a pivotal subject in medical research and public health policies in recent decades. Various studies have consistently affirmed the diverse benefits of regular physical exercise for the body and the mind [1,2,3]. From a physical health perspective, regular PA is crucial in preventing and managing a broad spectrum of chronic diseases. Research has demonstrated that physical exercise can markedly diminish the risks of cardiovascular diseases, type 2 diabetes, certain cancers, and osteoporosis [4]. In terms of mental health, the influence of PA is equally profound, with numerous studies underscoring its role in alleviating symptoms of depression and anxiety.

The advantages of PA extend to overall well-being [5]. Research indicates that physically active individuals report higher levels of life satisfaction, enhanced self-esteem, and a more remarkable ability to manage daily stress [6]. Recent investigations have begun to examine the benefits of PA beyond individual health, considering its impact on well-being, economic productivity, social cohesion, and environmental sustainability. Increasing PA at the country level could result in substantial healthcare savings by lowering the prevalence of chronic diseases [3].

This paper aims to explore the relationships between PA, physical inactivity (PI), a healthy life (HL), life expectancy (LE), and personal happiness (PH) among populations in European Union countries. The study uses artificial neural networks and cluster analysis to highlight the complex relationships between PA, PI, HL, LE, and PH. Acknowledging the unique characteristics of the EU, such as its diverse cultural, economic, and social landscapes, is essential in understanding how these factors differ from those in America, Asia, or Australia. This context enriches cross-country comparisons, allowing for a more nuanced analysis of the role of PA in enhancing public health and well-being in the EU. The goal is to underscore the importance of promoting an active lifestyle to improve health outcomes, identifying population groups with varying levels of PA and evaluating its influence.

This investigation has identified research gaps in quantifying the relationship between PA, PI, HL, LE, and PH at the EU level and the lack of comparable studies across EU countries on these variables. The unique aspects of this paper include the application of artificial neural network analysis for a comprehensive evaluation of the relationships between PA, PI, HL, LE, and PH; the use of cluster analysis to identify groups of countries based on their levels of PA and health; and a detailed comparative approach that highlights significant variations between EU countries. The research questions are as follows.

- RQ1. To what extent does PA influence HL years, LE, and PH?
- RQ2. Does PI significantly affect HL years, LE, and PH?
- RQ3. Do countries with a high level of PA record more HL years, higher LE, and superior levels of PH compared to countries where PA is at a low level?

These questions, which form the basis for the research hypotheses, guide our research, attempting to provide a deep understanding of the relationship between an active lifestyle and various aspects of HL and PH in the European context.

This paper is structured as follows. Section 1 introduces the research context, purpose, and gaps and presents the key research questions, followed by the aim of the study. Section 2 describes the materials and methods, detailing the procedures, literature review, hypotheses, instruments, data collection, and analysis techniques. Section 3 presents the research results, emphasizing the main findings. Section 4 discusses the results in relation to the existing literature, explores their broader implications, and suggests future research directions. Section 5 summarizes the study’s key conclusions.

## 2. Materials and Methods

### 2.1. Research Design

This research followed several stages to investigate the relationships between PA, PI, HL, LE, and PH in European Union countries. The first stage involved a literature review and hypothesis formulation, where we analyzed existing studies to identify knowledge gaps and establish our research hypotheses. The next stage entailed data collection, during which we gathered information from various statistical sources and relevant databases for our research variables. Subsequently, we proceeded to data processing and hypothesis testing, using advanced statistical methods to analyze the relationships between the variables. Finally, we presented and discussed the results in detail and formulated conclusions based on our findings, offering recommendations for future policies.

**Figure 1:**
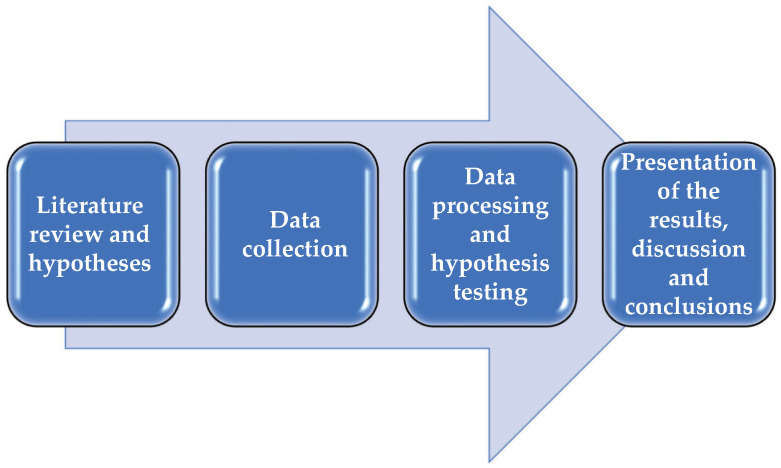
Research process stages. Source: authors’ design.

Figure 1 illustrates the research stages.

### 2.2. Literature Review and Hypotheses

Caspersen et al. define PA as “any bodily movement produced by skeletal muscles that results in energy expenditure” ([7], p. 126). PA plays a decisive role in managing body weight, thus preventing obesity, a risk factor for numerous chronic conditions, including type 2 diabetes, hypertension, and cardiovascular diseases [8]. Regular exercise improves flexibility, balance, and coordination, which is essential in preventing accidents and falls, particularly for older adults [9]. Regular PA helps to maintain bone and joint health, reducing the risk of osteoporosis and other degenerative diseases of the musculoskeletal system.

People can integrate various forms of PA into their daily lives and not only traditional sports or structured exercises that contribute to physical and mental health [10]. Occupational activities, such as physical work performed at the workplace, can significantly maintain good health [11,12].

PA positively impacts mental health, reducing symptoms of depression and anxiety, increasing PH, and improving overall well-being [13]. Several studies have confirmed the physiological effects of physical exercise, showing that PA can significantly and positively impact mental health [14,15,16,17,18,19]. Physical exercises contribute to developing a daily routine and structure, improving sleep quality and positively affecting mental health [20]. A regular exercise program can provide control and predictability, essential in reducing stress and anxiety [21,22].

Building emotional resilience is another effect of PA, providing individuals with better mechanisms to cope with daily stress and challenges. Regular participation in physical exercise can contribute to developing a more positive mindset, as PA promotes a sense of achievement and self-efficacy [23]. This fact is a determinant in increasing self-confidence and self-esteem, fundamental elements of psychological well-being [13]. Adequate physical activity positively influences individuals’ subjective well-being and contributes to developing a more positive psychological perspective, improving social relationships, and increasing overall life satisfaction [19,24]. Integrating physical exercise into daily routines is essential in maintaining a healthy balance between body and mind, profoundly impacting quality of life [25].

Socially, participation in PA, such as team sports or fitness classes, can encourage social interaction and the formation of new relationships, thus contributing to developing social skills and increasing the sense of belonging to a community [26]. PA brings evident physical benefits and profoundly impacts social health, making it important in maintaining a healthy and balanced life [13,27,28]. Therefore, integrating PA into daily routines is an effective strategy to ensure robust mental health and PH and enhance overall well-being. This social aspect is critical for general well-being, as social support and interaction with others constitute significant determinants of mental and emotional health [10,29].

Physical exercise and sports are often associated with improved cognitive performance and concentration. Regular PA can improve memory, attention, and learning capacity, thus significantly benefiting physical health and intellectual performance [30]. Regular PA improves physical health and profoundly impacts psychological well-being and overall PH [29,31,32,33]. Through a holistic approach to health, which includes both psychological and social aspects, individuals can aspire to a society where they not only live longer but live better, with a deep sense of fulfillment and satisfaction [34].

Studies have shown that people who regularly engage in PA tend to report higher levels of energy, improved mood, and an increased ability to cope with daily stress [23]. These benefits directly translate into improving the perception of quality of life [35]. Involvement in PA can provide a sense of personal achievement and progress, which fuels self-esteem and self-confidence [36]. Physically active individuals often report greater ease in performing daily tasks, from climbing stairs to carrying groceries or playing with children. This increase in functional capacity significantly contributes to feelings of independence and autonomy, which are significant factors in PH [37].

PA improves the body’s effort capacity and endurance, allowing the individual to perform daily activities more efficiently and have a more active life. Improved cardiovascular function means that the heart and lungs work more efficiently, transporting oxygen and nutrients throughout the body [3]. This fact proves essential in preventing heart disease and stroke, which rank among the leading causes of death globally. PA also positively impacts other health markers, such as cholesterol and blood sugar levels, which are decisive in preventing metabolic diseases and ensuring a HL [5]. However, leisure-time PA is also associated with higher risks of malignant melanoma and prostate cancer. This may be explained by the fact that outdoor activities expose the skin to ultraviolet radiation, thus increasing the risk of skin cancer [38]. Furthermore, some studies suggest that intense physical exercise can influence hormone levels, which may impact the risk of prostate cancer.

This paper proposes Hypothesis H1 to test the relationship between PA, HL, LE, and PH.

**Hypothesis** **H1.** *PA has a significant positive impact on HL years, LE, and PH.*

PI and a sedentary lifestyle have severe consequences for cardiovascular health, increasing the risks of hypertension, coronary heart disease, and stroke [39]. Physically inactive individuals face a higher risk of developing obesity, which in turn serves as a risk factor for numerous other chronic conditions, including heart disease, diabetes, and certain types of cancer [40]. Excess weight can lead to orthopedic problems, such as osteoarthritis, and negatively affect mental health by lowering self-esteem and increasing the risk of depression. People who do not engage in regular physical activity may experience a deterioration in their overall quality of life [30].

The negative impact of PI extends beyond the individual, affecting families and society as a whole. Families may experience an increased financial burden due to the medical costs associated with treating inactivity-related diseases and obesity. Moreover, workplace productivity can suffer, as physically inactive employees have a higher likelihood of experiencing chronic diseases and taking frequent medical leave [12]. At a societal level, PI contributes to rising costs in public health systems due to the need to treat more patients with chronic conditions. Promoting an active lifestyle through public policies and community programs can significantly reduce these costs and improve the population’s overall health. PA plays a central role in maintaining mental and emotional health [10] and promotes better weight management, reduces inflammation, and enhances immune system function.

This paper proposes Hypothesis H2 to investigate the relationship between PI, HL, LE, and PH.

**Hypothesis** **H2.** *PI has a significant adverse impact on HL years, LE, and PH.*

The significant disparities in leisure-time PA (LTPA) levels among different welfare state models in Europe reflect the profound impact that social, economic, and cultural structures have on health-related behaviors [41]. These differences represent more than mere statistics; they manifest concretely how public policies, infrastructure, and societal values shape citizens’ lives and choices [9].

The Nordic welfare model, characterized by substantial investments in public services, education, and health promotion programs, creates an environment conducive to PA. These countries tend to have well-developed infrastructures for outdoor activities, such as bicycle paths, parks, and sports facilities accessible to the general public. The Nordic culture strongly values time spent in nature and PA, which is reflected in the high rate of participation in LTPA [10,41].

In contrast, the Mediterranean model, although recognized for other positive aspects of lifestyle (such as the Mediterranean diet), appears less effective in promoting structured LTPA [42]. This situation may stem from a combination of factors, including cultural differences in the perception of active aging, less developed infrastructure for PA, and possibly the greater reliance on family networks for elderly care, which may limit the opportunities or motivation for LTPA.

The importance of perceiving opportunities to engage in LTPA in the residential area underscores the significant role of urban planning and local policies in promoting an active lifestyle [9]. The availability and accessibility of green spaces, sports facilities, and community PA programs can significantly influence participation levels. This suggests that community-level interventions can substantially impact LTPA levels [41].

Moreover, these differences raise important questions about health equity at the European level. If opportunities for LTPA are distributed unequally across different regions and welfare state models, this may contribute to broader inequalities in HL and PH for the elderly population [11]. Addressing these disparities may require a coordinated approach at the European level, considering specific cultural and economic contexts but aiming to increase the overall PA levels across all regions [42].

Another critical aspect involves how these differences in LTPA translate into long-term health outcomes. Nordic countries tend to have a higher LE, which researchers may partially attribute to higher levels of PA [11]. Understanding and replicating the success factors from the Nordic model could offer valuable insights to improve the population’s health across Europe [10].

This paper proposes Hypothesis H3 to investigate the relationship between physical PA, HL, LE, and PH.

**Hypothesis** **H3.** *European Union countries with a high level of PA have higher HL years, LE, and PH levels.*

### 2.3. Selected Variables

To evaluate the relationships between PA, HL, LE, and PH, we selected the following variables: healthy life years at birth (HLYB), life expectancy at birth (LRB), self-reported happiness levels, and variables related to physical exercise and PA.

PA encompasses various movements and activities that form part of daily life: occupational PA, transport-related PA, household PA, and LTPA. This comprehensive approach to PA underscores the importance of adopting an active lifestyle in all aspects of life and not only during time dedicated to planned exercises [41,42,43,44,45]. Acknowledging and promoting all of these forms of PA can contribute to creating healthier and more active societies where people value and encourage movement in all aspects of daily life.

This paper uses data from the Eurobarometer published by the European Commission [11] to describe the PA variables. The Eurobarometer’s approach to assessing the PA of the European population, with a focus on LTPA, offers a valuable but limited perspective on overall PA levels. This focus on LTPA reflects the growing importance given to leisure time and recreational activities in modern societies but also raises important questions about how we measure and understand PA in a broader context. Including structured sports exercises and less formal leisure activities in the definition of LTPA represents an essential step in recognizing the diversity of the ways in which people can be physically active. This more inclusive approach can better capture the reality of daily life for many Europeans, where activities such as gardening or dancing may constitute a significant part of their total PA. Table 1 shows the research variables selected.

### 2.4. Methods

We employed two methods for data analysis: artificial neural network analysis and cluster analysis. Artificial neural network analysis allowed us to evaluate the complex relationships between our variables, providing a detailed perspective on how PA and PI influence HL, LE, and PH. This method identifies the nonlinear patterns and interactions between variables [48]. Cluster analysis grouped the European Union countries based on their levels of PA, HL, LE, and PH [49]. This method highlighted the differences and similarities between the countries, allowing us to identify groups with similar characteristics.

## 3. Results

This section provides an overview of the research findings, structured around the three main hypotheses (H1, H2, and H3) and their corresponding research questions (RQ1, RQ2, and RQ3). The results highlight the relationships between PA, PI, and key health indicators: HL, LE, and PH. Each hypothesis is examined through different analytical techniques, providing insights into the influence of PA and PI on health outcomes across EU countries.

### 3.1. Results Related to Hypothesis H1

Investigating Hypothesis H1 involved using a multilayer perceptron (MLP) in an artificial neural network analysis. The results of this research help us to answer RQ1: To what extent does PA influence HL years, LE, and PH? The analysis provides insightful correlations and highlights the significant impact that PA has on these health-related indicators.

**Figure 2:**
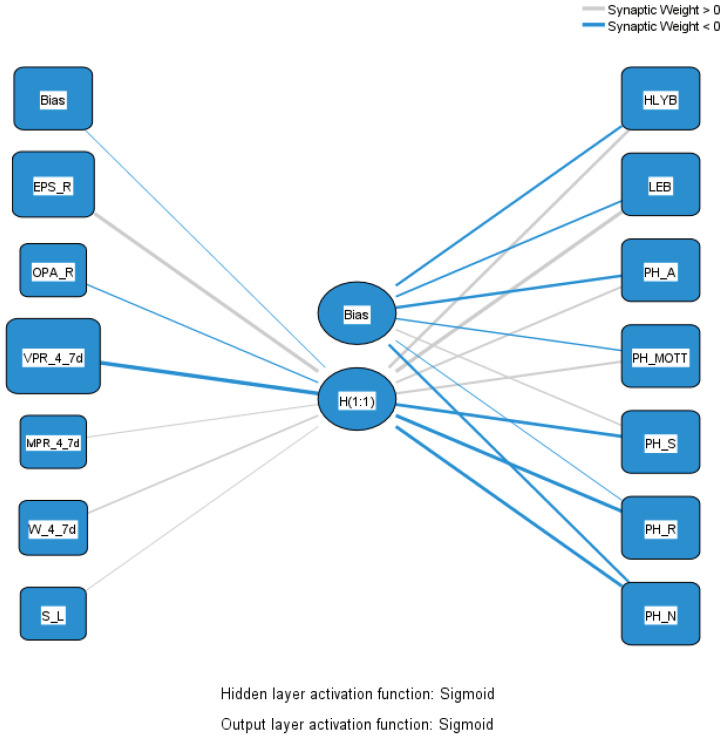
The MLP model concerning PA, HL, LE, and PH relationships. Source: authors’ design using SPSS v.27.

The MLP’s input layer comprises variables describing PA: exercise or play sports—regularly; other physical activity—regularly; vigorous physical activity—4–7 days; moderate physical activity—4–7 days; walk for at least 10 min—4–7 days; time spent sitting on a usual day—2 h 30 min or less. The output layer includes variables representing HL, LE, and PH: healthy life years in absolute value at birth; life expectancy in absolute value at birth; persons being happy in the last four weeks—always; persons being happy in the last four weeks—most of the time; persons being happy in the last four weeks—sometimes; persons being happy in the last four weeks—rarely; persons being happy in the last four weeks—never. An intermediary hidden layer, representing the active lifestyle adopted by citizens of each EU country, exists between these layers. The relationship diagram in Figure 2 illustrates the connections between the input variables, the hidden layer, and the output variables, highlighting the model’s complexity and the interactions between PA, HL, LE, and PH.

In Figure 2, the connections between nodes are represented by lines indicating synaptic weights, with gray lines denoting positive weights (>0) and blue lines representing negative weights (<0). The model employs sigmoid activation functions for both the hidden and output layers. This MLP structure is designed to process input data through the hidden layer and produce multiple outputs, suggesting its capability for complex predictions or classifications based on various input factors.

Table 2 shows the predictors for the input and hidden layers and the importance of the input variables.

PA significantly impacts HL, LE, and PH. The model highlights a robust positive influence between PA and LEB (1.662), suggesting that individuals who frequently participate in PA tend to have augmented longevity. Furthermore, a positive influence exists between PA and HLYB, with a weight of 1.008, indicating that PA not only extends life but also improves its quality by increasing the period during which a person enjoys good health.

Regarding PH, various relationships emerge between PA and various reported happiness levels. A positive influence appears with the feeling of being “always” and “most of the time” happy, while negative influences are observed with the feeling of being “sometimes”, “rarely”, and “never” happy. These results suggest that regular PA tends to increase the frequency of PH and decrease the frequency of unhappiness or indifference.

Different types of PA hold varying importance in the model. Regular exercise or sports practice has the highest positive weight, strongly influencing HL, LE, and PH. Conversely, vigorous PA performed 4–7 days a week shows a significant negative weight, possibly suggesting that frequent intense exercise may have adverse effects, potentially due to overexertion or an increased risk of injury or various diseases. Moderate PA and walking have positive weights, emphasizing the benefits of regular PA, even at moderate intensities.

The MLP model provides solid evidence that regular and moderate PA has a significant positive impact on HL, LE, and PH, confirming the validity of Hypothesis H1. These results underscore the importance of incorporating PA into daily routines to improve HL, LE, and PH, highlighting the complexity of the relationships between different types and intensities of PA and various aspects of health and happiness.

### 3.2. Results Related to Hypothesis H2

**Figure 3:**
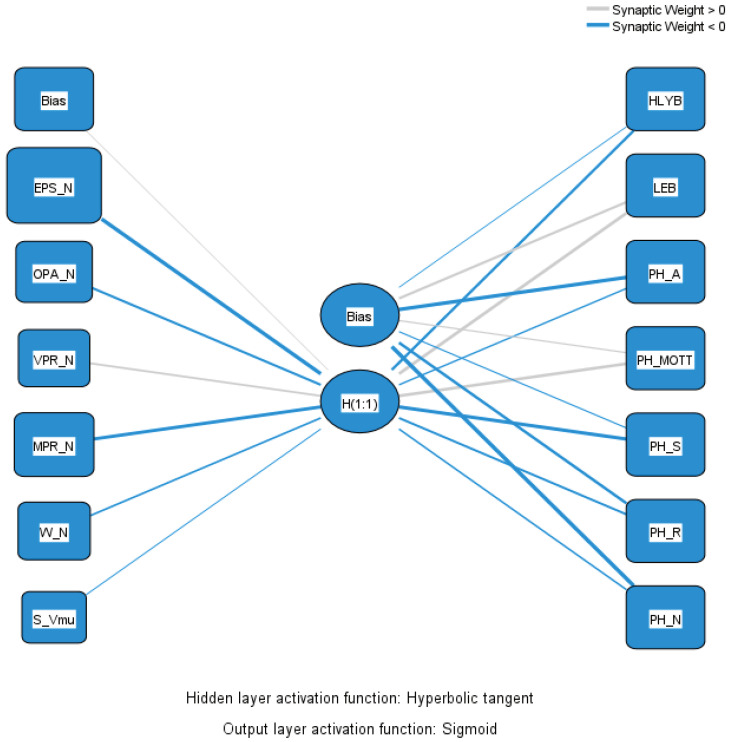
The MLP model concerning PI, HL, LE, and PH relationships. Source: authors’ design using SPSS v.27.

Investigating Hypothesis H2 involved using another MLP in a network analysis. The results of this research help us to answer RQ2: Does PI significantly affect HL years, LE, and PH? The findings reveal the extent to which PI influences these indicators, providing a clearer understanding of its role in shaping health outcomes. The MLP’s input layer comprises variables describing PI: exercise or play sports—never; other physical activity—never; vigorous physical activity—never; moderate physical activity—never; walk for at least 10 min—never; time spent sitting on a usual day—8 h 31 min or more. The output layer includes variables representing HL, LE, and PH: healthy life years in absolute value at birth; life expectancy in absolute value at birth; persons being happy in the last four weeks—always; persons being happy in the last four weeks—most of the time; persons being happy in the last four weeks—sometimes; persons being happy in the last four weeks—rarely; persons being happy in the last four weeks—never. An intermediary hidden layer, representing the inactive lifestyle adopted by citizens of each EU country, occurs between these layers. The hyperbolic tangent activation function for the hidden layer and the sigmoid activation function for the output layers facilitate the capture of nonlinear relationships between the variables. The relationship diagram in Figure 3 illustrates the connections between the input variables, the hidden layer, and the output variables, highlighting the model’s complexity and the interactions between PI, HL, LE, and PH.

In Figure 3, the connections between nodes are represented by lines indicating synaptic weights, with gray lines denoting positive weights (>0) and blue lines representing negative weights (<0). The model employs sigmoid activation functions for both the hidden and output layers.

Table 3 shows the predictors for the input and hidden layers and the importance of the input variables.

Analyzing the data reveals that PI has a complex and generally negative influence on various aspects of HL, LE, and PH. Firstly, the absence of exercise and sports (EPS_N) has the most significant negative impact, with a weight of −0.949 and normalized importance of 100%. This fact suggests that the complete absence of structured PA is the most detrimental factor to HL, LE, and PH. Following this, the lack of moderate PA (MPR_N) has a weight of −0.598 and normalized importance of 53.3%, indicating that the absence of medium-intensity physical activity also has a significant negative effect. The absence of vigorous PA (VPR_N) has a positive weight of 0.345, suggesting that the absence of highly demanding exercise is not necessarily harmful. This situation could be interpreted in the context of the risks associated with overexertion or injuries caused by overly intense PA.

Prolonged sitting (S_Vmu) has a relatively small negative weight (−0.056). However, this should not minimize the importance of reducing sedentary behavior, particularly since other studies have shown the negative effects of prolonged sedentary behavior [39,40]. Regarding the impact on HL and LE, PI negatively influences healthy life years at birth (HLYB), with a weight of −0.468. In contrast, it positively influences LEB, with a weight of 0.585. This apparent contradiction might suggest that although PI does not necessarily reduce life span, it negatively affects life quality and the number of years lived in optimal health.

Regarding PH, the model shows that PI negatively impacts the feeling of being “always” happy (−0.219) and “sometimes” happy (−0.664), while positively influencing the feeling of being happy “most of the time” (0.570). This fact suggests that PI may lead to more significant fluctuations in emotional states, reducing periods of constant PH. It is essential to note the strongly negative influence of PI on the feeling of never being happy (−0.219), which might indicate that a deficiency in PA increases the risk of anxiety and depression.

In conclusion, this MLP model highlights the complex negative impact of PI on HL, LE, and PH, confirming the validity of Hypothesis H2. It underscores the importance of maintaining an adequate PA level to improve one’s physical and mental health.

### 3.3. Results Related to Hypothesis H3

Investigating Hypothesis H3 involved a cluster analysis, which grouped the EU countries based on their PA, HL, LE, and PH levels. The results of this research help us to answer RQ3: Do countries with a high level of PA record more HL years, higher LE, and superior levels of PH compared to countries where PA is at a low level? The analysis highlights significant patterns, demonstrating that countries with higher PA levels generally tend to achieve better health outcomes.

The Ward method minimizes intra-cluster variance, thus ensuring the formation of homogeneous groups. The Ward method is a hierarchical agglomerative technique that minimizes the total variance within each cluster [50]. At each agglomeration stage, two clusters are joined to minimize the sum of squared differences between points in the cluster and the cluster center. As a distance measure, this study used the squared Euclidean distance, a variant of the Euclidean distance that measures the square of the direct distance between two points in a multidimensional space.

**Figure 4:**
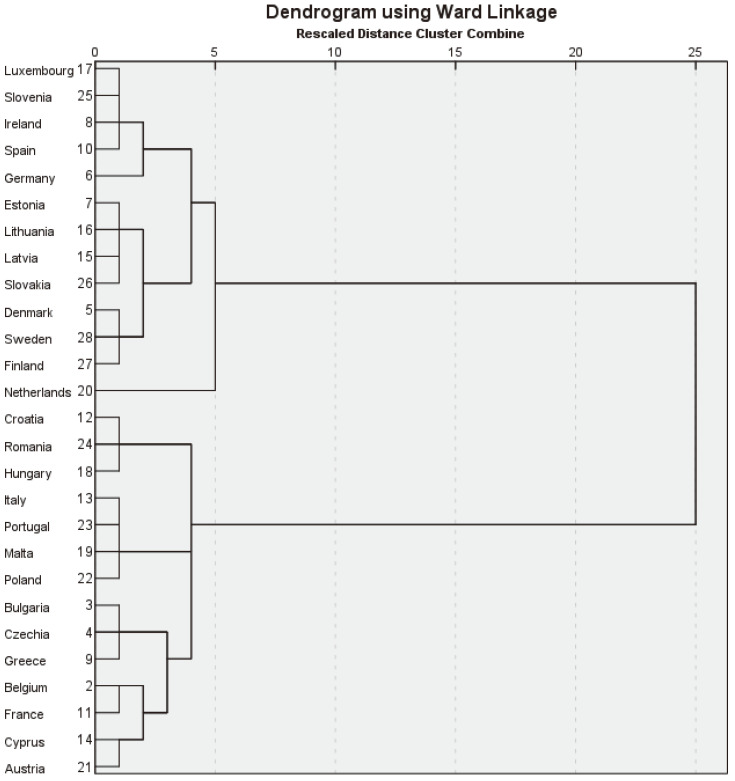
Dendrogram. Source: authors’ design using SPSS v.27.

This study identified distinct groups of EU countries exhibiting similar characteristics regarding PA, HL, LE, and PH levels. This fact gave a deeper understanding of how these countries stand concerning these indicators and highlighted significant variations among them. Figure 4 shows the dendrogram resulting from the cluster analysis.

Table 4 presents data on PA, HL, LE, and PH within EU countries, which are grouped into two clusters.

A careful analysis of the data from Table 4 reveals significant differences between the two groups of countries. Cluster A, which includes countries such as Luxembourg, Slovenia, Ireland, and Germany, generally shows higher values for the PA and PH indicators. The average HLYB for this cluster is 60.4 years, slightly below the EU average of 62.1 years. However, the LEB is 80.3 years, above the EU average of 79.9 years. Regarding PH, the countries in Cluster A have a higher average number of people who report being “always” happy and “most of the time” happy (63.5%) compared to the EU average (61.4%). These countries have lower rates of people reporting being “sometimes”, “rarely”, or “never” happy.

PA is significantly higher in Cluster A. For example, the average for regular exercise or sports practice (EPS_R) is 10.2%, compared to the EU average of 7.6%. Similarly, other regular physical activities (OPA_R) have an average of 23% in Cluster A, compared to the EU average of 16.2%. Cluster B, which includes countries such as Croatia, Romania, Italy, and France, presents a mixed picture. The average HLYB is higher in Cluster B (63.7 years) than in Cluster A and the EU average. However, the LEB (79.6 years) is slightly lower than the EU average and that in Cluster A. Cluster B has a higher percentage of people who declare themselves happy “always” (11.4%). Still, fewer people declare themselves happy “most of the time” (48.1%) compared to Cluster A (53.3%). PA in Cluster B is generally lower than in Cluster A and below the EU average, with an average of only 5.2% for EPS_R and 9.9% for OPA_R.

In conclusion, the results presented in Table 4 provide a comprehensive overview of the state of PA, HL, LE, and PH in the EU, highlighting the significant differences between the two clusters of countries. These findings confirm the validity of Hypothesis H3, suggesting that countries with higher PA levels generally show better indicators of HL, LE, and PH. The data emphasize the importance of promoting PA at the individual and national levels to improve EU citizens’ HL, LE, and PH. The clustering of countries highlights the need for customized strategies to address each country’s specific challenges and opportunities, recognizing the diversity and complexity of the factors influencing PA, HL, LE, and PH in the EU.

## 4. Discussion

This paper investigates the relationships between PA, HL, LE, and PH indicators, testing and validating three main hypotheses. Hypothesis H1 explored the relationships between PA, PI, HL, LE, and PH, suggesting that it should become part of the daily routine, making people more consistent in practicing exercise. The analysis revealed that PA, mainly moderate activity, has a significant positive impact on HL, LE, and PH, confirming the validity of Hypothesis H1. Previous studies in the literature support these findings. The literature highlights that moderate PA effectively improves subjective well-being and prevents mental health problems, confirming our findings. Shennar-Golan [51] emphasizes that moderate PA, being more accessible and sustainable, proves preferable for most people compared to high-intensity exercise, which may be too demanding. Regular PA significantly contributes to preventing and treating a wide range of diseases while improving mental health and overall quality of life [52,53].

PA also plays a decisive role in stress management by reducing cortisol levels, the stress hormone, leading to a general state of relaxation. Wang and Wang [54] mention that people who engage in regular PA report a more remarkable ability to cope with stressful situations and more excellent stress resistance. Sleep quality also reflects this action, where physical exercise helps to regulate the circadian rhythm and promotes more profound and restful sleep.

Regular PA can also release accumulated tension, preventing stress buildup and reducing the risk of burnout and other problems associated with chronic stress, as shown by the study of Kiecolt-Glaser et al. [21]. In addition to reducing stress, PA promotes a sense of achievement and control, thus improving self-confidence and self-esteem, essential aspects for interpersonal relationships and overall life satisfaction, as mentioned by Gualdi-Russo and Zaccagni [52].

Our findings align with recent studies concerning moderate PA’s positive effects on subjective well-being. Yang et al. [19] emphasize that moderate-intensity exercises, such as walking, light cycling, or swimming, allow the body to benefit from the advantages of movement without subjecting it to excessive stress, thus leading to a general state of well-being and an increased sense of control over one’s life. White et al. [55] add that these moderate exercises reduce symptoms of anxiety and depression, contributing to greater mental resilience and a more positive outlook on life, with the psychological benefits being particularly significant in improving quality of life.

Moderate PA proves indispensable in preventing and treating chronic diseases and improving mental health and quality of life, contributing to increased subjective well-being [56]. Our results demonstrate that PA contributes to improved PH. This feeling of PH is also reflected in one’s mental state, contributing to a better mood and a reduction in stress and anxiety levels [57,58,59,60]. In the context of healthy aging, outdoor sports prove particularly beneficial for older adults [61]. These activities offer movement and physical exercise opportunities, socialization, and interactions with nature, which can positively impact mental and emotional health. Studies have shown that older adults who engage in outdoor sports maintain better physical function and are less susceptible to developing age-related conditions such as arthritis and osteoporosis [62].

The investigation of Hypothesis H2 involved using an MLP to analyze the impact of PI on HL, LE, and PH in the EU. The analysis showed that a lack of PA has the most significant adverse effects on HL and PH, confirming the validity of Hypothesis H2. Previous studies in the literature support these findings. PI has substantial consequences for cardiovascular health, orthopedic problems, and mental health, lowering self-esteem and increasing the risk of depression [39]. At a societal level, PI contributes to rising costs in public health systems due to the need to treat more patients with chronic conditions [12]. The MLP model demonstrated that the complete absence of structured PA proves to be the most detrimental factor for HL, LE, and PH. In contrast, even moderate levels of PA can have significant benefits. Avoiding complete inactivity, including in the workplace [63,64], plays a decisive role in maintaining good health and a high level of PH.

The cluster analysis highlighted significant differences between the two groups of EU countries regarding PA, HL, LE, and PH, confirming the validity of Hypothesis H3. Cluster A, which includes countries such as Luxembourg, Slovenia, Ireland, and Germany, showed high values for the HL and PH indicators. Countries in Cluster A have a higher percentage of people who declare themselves happy “always” and “most of the time” (63.5%) compared to the EU average (61.4%). PA appears significantly higher in Cluster A than the EU average. In contrast, Cluster B, which includes countries such as Croatia, Romania, Italy, and France, presents a mixed picture. Although some indicators, such as LEB, are close to the EU average, the PA and PH levels are generally lower. The average for regular exercise or sports practice (EPS_R) in Cluster B is well below the EU average. This fact suggests that countries in Cluster B might face more significant challenges in promoting PA and PH among their populations. However, the diversity within this cluster indicates that some countries might be performing better than others in certain areas. These observations suggest that although Cluster B has lower rates of PA, it presents a higher average number of HL years. This finding indicates that other factors, such as diet, cultural differences, and climate, are essential in determining HL, LE, and PH. These findings support previous research highlighting the significant impact of social, economic, and cultural structures on health-related behaviors, alongside PA [41].

### 4.1. Theoretical Implications

The findings of this study emphasize the importance of promoting regular PA, particularly of moderate intensity, as a determinant strategy to enhance quality of life and prevent health issues. The study highlighted a robust positive link between PA, HL, and LE, emphasizing that individuals who engage in regular physical exercises tend to live longer and enjoy better quality of life. Moreover, this research underscores the importance of moderate PA, which has proven more sustainable and accessible for most of the population compared to high-intensity exercises. This finding suggests that moderate PA should receive priority in public health recommendations. This research contributes to the existing literature by highlighting the psychological effects of PA on PH. The study offers solid empirical evidence regarding the role of PA in promoting PH. The empirical models have shown that PA is associated with a higher frequency of states of happiness and a reduction in states of unhappiness or indifference, emphasizing the importance of integrating physical exercise into daily life to improve one’s quality of life. These theoretical implications not only broaden the understanding of the benefits of PA but also provide a foundation for the development of more effective policies in public health and health psychology.

### 4.2. Practical Implications

The results of this study suggest that public health programs should actively encourage moderate PA, which has proven to be more sustainable and accessible across various age groups. This approach can involve implementing information and education campaigns that highlight the benefits of activities such as walking, light cycling, and other forms of moderate exercise. Policies that enhance access to public spaces dedicated to PA—such as parks, bike lanes, and community sports facilities—can foster increased participation in physical activity among the population.

The findings underscore the importance of integrating regular exercise into individuals’ daily routines to improve PH. Mental health professionals can use this information to recommend PA as part of treatment plans for patients experiencing depression, anxiety, and other mood disorders. Workplace wellness programs may benefit from incorporating regular exercise sessions to alleviate stress and enhance employee productivity. Educators and community leaders can also leverage these findings to create initiatives promoting PA among youth, which can help to mitigate the risks of obesity and other health issues associated with sedentary lifestyles. Schools should consider increasing the number of physical education classes and organizing sports events to encourage active participation among students.

The results also indicate that PA positively influences happiness and well-being, suggesting that interventions promoting physical activity could yield long-term benefits for overall life satisfaction. This result is particularly relevant for aging populations, where maintaining good health and a positive outlook becomes increasingly critical. Programs customized to the needs and abilities of seniors can promote an active and healthy lifestyle.

The significant variability in PA patterns across different countries highlights the necessity for customized and multidimensional approaches to promoting physical activity among seniors. This approach involves not only public health policies but also a re-evaluation of urban design, leisure time organization, and societal attitudes toward PA. Europe can make significant strides toward fostering a healthier and more active senior population, thereby enhancing quality of life and reducing long-term healthcare costs.

### 4.3. Limitations

Certain limitations in our study might affect the interpretation and generalization of the results. One major limitation is the focus on aggregated country-level data, which can mask significant regional or individual variations. Socioeconomic, cultural, and environmental factors specific to each region or locality can significantly influence PA levels, HL, and LE—aspects not fully captured in a national-level analysis.

The cross-sectional nature of the data used limits our ability to establish transparent causal relationships between PA, PI, HL, LE, and PH. Although the statistical models used suggest robust associations, they cannot definitively demonstrate causality, which would require long-term longitudinal studies. Other factors were omitted, despite efforts to include a wide range of relevant variables in the analysis, due to the scarcity of data. Factors like diet, genetic factors, healthcare system quality, and public health policies may significantly influence health and well-being outcomes. However, they could not be included in our models due to data limitations.

The exclusive focus on LTPA from the Eurobarometer data [11] used in this study also imposes significant limitations. The Eurobarometer tends to underestimate the total PA levels of individuals who have physically active jobs or exhibit substantial transport or household PA levels. Particularly in the European context, where considerable variations exist in lifestyles and occupational structures between different countries and regions, a complete picture of PA would require the consideration of all domains of PA.

### 4.4. Further Research

Due to these limitations, future research could make valuable contributions by conducting longitudinal studies that track cohorts of individuals over extended periods. Such studies would enable researchers to observe the long-term effects of PA on various dimensions of HL, LE, and PH.

Future research could emphasize how different types of PA—such as aerobic exercises, strength training, and flexibility workouts—affect these health dimensions in diverse populations. Understanding the nuances of these relationships could provide insights into the mechanisms by which PA influences health outcomes. Furthermore, interdisciplinary studies that integrate perspectives from medicine, psychology, sociology, and public health policy could offer a more integrated understanding of these complex interactions. Through collaboration across disciplines, researchers can better address the multifaceted nature of PA and its impact on health. This approach could uncover social determinants of health that influence PA participation, as well as psychological barriers that prevent individuals from engaging in regular physical activity.

Future studies should explore the demographic variables that influence physical activity (PA) levels, particularly age, as children, adolescents, adults, and seniors show distinct PA patterns. Factors such as gender, socioeconomic status, and ethnicity also play critical roles in access to and engagement in PA. The impact of the COVID-19 pandemic on these dynamics requires investigation, as it may have changed the established PA behaviors across various populations. Studies should adopt an ecological perspective, utilizing frameworks like Bronfenbrenner’s Ecological Systems Theory to examine how individual behaviors are shaped by broader environmental contexts and social systems [65]. Addressing these variables will enhance our understanding of PA and its determinants, leading to more effective strategies to promote active lifestyles among diverse populations.

Cross-country characteristics that influence physical activity and inactivity can be another focus of future studies, examining barriers related to factors such as the country, race, age, culture, occupation, disease, policy, social context, and personal tendencies. This approach can involve qualitative studies that explore personal attitudes and beliefs about exercise, as well as the influence of community resources and support systems. By addressing these diverse barriers, future research can contribute to the development of targeted interventions and policies that promote active lifestyles across various demographics.

## 5. Conclusions

In the modern era, characterized by increasingly sedentary lifestyles and the rising prevalence of chronic diseases, understanding the role of PA in promoting health and quality of life becomes central. The main findings of our research confirmed and extended existing knowledge in the field. We proved that regular PA has a significant positive relationship with HL, LE, and PH levels. Moreover, we observed that moderate PA offers the most accessible and sustainable benefits for most of the population, being more accessible for integration into daily routines and leading to greater consistency in exercise practices. The analysis also highlighted the negative consequences of PI, confirming that a lack of exercise and a sedentary lifestyle have detrimental effects on HL and LE. These findings underscore the critical importance of policies and programs to reduce sedentary behaviors and promote an active lifestyle.

This research provides robust evidence for the fundamental role of PA in enhancing population health and well-being. The findings highlight the necessity for comprehensive public policies that facilitate and encourage participation in regular PA, customized to the diverse needs and preferences of the population. Furthermore, this study contributes to the literature by demonstrating the complex connections between PA and various health outcomes, offering a valuable framework for future research. At the same time, the results open new research directions, suggesting the need for more in-depth studies to understand better the mechanisms through which PA influences various aspects of health and well-being and to develop more effective strategies to promote an active lifestyle in modern societies.

## Figures and Tables

**Table 1:** Selected variables and measures.

Variable	Dataset	Measure	Source
HLYB	Healthy life years in absolute value at birth	Percentage	[46]
LEB	Life expectancy in absolute value at birth	Percentage	[46]
PH_A	Persons being happy in the last four weeks—Always	Percentage	[47]
PH_MOTT	Persons being happy in the last four weeks—Most of the time	Percentage	[47]
PH_S	Persons being happy in the last four weeks—Sometimes	Percentage	[47]
PH_R	Persons being happy in the last four weeks—Rarely	Percentage	[47]
PH_N	Persons being happy in the last four weeks—Never	Percentage	[47]
PH_DK	Persons being happy in the last four weeks—I don’t know	Percentage	[47]
EPS_R	Exercise or play sports—Regularly	Percentage	[11]
EPS_WSR	Exercise or play sports—With some regularity	Percentage	[11]
EPS_S	Exercise or play sports—Seldom	Percentage	[11]
EPS_N	Exercise or play sports—Never	Percentage	[11]
EPS_DK	Exercise or play sports—I don’t know	Percentage	[11]
OPA_R	Other physical activity—Regularly	Percentage	[11]
OPA_WSR	Other physical activity—With some regularity	Percentage	[11]
OPA_S	Other physical activity—Seldom	Percentage	[11]
OPA_N	Other physical activity—Never	Percentage	[11]
OPA_DK	Other physical activity—I don’t know	Percentage	[11]
VPR_1-3d	Vigorous physical activity—1–3 days	Percentage	[11]
VPR_4-7d	Vigorous physical activity—4–7 days	Percentage	[11]
VPR_N	Vigorous physical activity—Never	Percentage	[11]
VPR_DK	Vigorous physical activity—I don’t know	Percentage	[11]
MPR_1-3d	Moderate physical activity—1–3 days	Percentage	[11]
MPR_4-7d	Moderate physical activity—4–7 days	Percentage	[11]
MPR_N	Moderate physical activity—Never	Percentage	[11]
MPR_DK	Moderate physical activity—I don’t know	Percentage	[11]
W_1-3d	Walk for at least 10 min—1–3 days	Percentage	[11]
W_4-7d	Walk for at least 10 min—4–7 days	Percentage	[11]
W_N	Walk for at least 10 min—Never	Percentage	[11]
W_DK	Walk for at least 10 min—I don’t know	Percentage	[11]
S_L	Time spent sitting on a usual day—2 h 30 min or less	Percentage	[11]
S_Mo	Time spent sitting on a usual day—2 h 31 min to 5 h 30 min	Percentage	[11]
S_Mu	Time spent sitting on a usual day—5 h 31 min to 8 h 30 min	Percentage	[11]
S_Vmu	Time spent sitting on a usual day—8 h 31 min or more	Percentage	[11]
S_DK	Time spent sitting on a usual day—I don’t know	Percentage	[11]

Source: Developed by the authors based on [11,46,47].

**Table 2:** MLP model predictors for PA.

Predictor		Predicted									
		Hidden Layer 1	Output Layer							Importance	
		H(1:1)	HLYB	LEB	PH_A	PH_MOTT	PH_S	PH_R	PH_N	Importance	Normalized Importance
Input Layer	(Bias)	−0.029									
	EPS_R	1.243								0.290	62.1%
	OPA_R	−0.241								0.057	12.1%
	VPR_4_7d	−2.130								0.466	100.0%
	MPR_4_7d	0.228								0.053	11.4%
	W_4_7d	0.360								0.086	18.5%
	S_L	0.204								0.048	10.2%
Hidden Layer 1	(Bias)		−0.772	−0.405	−0.956	−0.0277	0.316	−0.042	−0.826		
	H(1:1)		1.008	1.662	0.467	0.748	−1.057	−1.128	−1.117		

Source: authors’ design using SPSS v.27.

**Table 3:** MLP model predictors for PI.

Predictor		Predicted									
		Hidden Layer 1	Output Layer							Importance	
		H(1:1)	HLYB	LEB	PH_A	PH_MOTT	PH_S	PH_R	PH_N	Importance	Normalized Importance
Input Layer	(Bias)	0.014									
	EPS_N	−0.949								0.389	100.0%
	OPA_N	−0.435								0.167	42.9%
	VPR_N	0.345								0.105	26.9%
	MPR_N	−0.598								0.207	53.3%
	W_N	−0.314								0.114	29.4%
	S_Vmu	−0.056								0.018	4.5%
Hidden Layer 1	(Bias)		−0.029	0.456	−0.757	0.129	−0.127	−0.471	−1.387		
	H(1:1)		−0.468	0.585	−0.219	0.570	−0.664	−0.346	−0.219		

Source: authors’ design using SPSS v.27.

**Table 4:** Clusters.

	HLYB	LEB	PH_A	PH_MOTT	PH_S	PH_R	PH_N	EPS_R	OPA_R	VPR_4_7d	MPR_4_7d	W_4_7d	S_L
Luxembourg	60.2	83.0	14.2	62.7	17.3	4.2	0.7	13	22	25	30	66	20
Slovenia	66.7	81.3	10.5	55.8	24.5	7.6	1.1	11	18	20	31	69	19
Ireland	66.0	82.6	7.9	65.5	22.0	3.5	1.1	13	10	26	35	65	16
Spain	61.2	83.2	22.8	44.5	26.0	4.8	1.9	11	10	25	30	77	10
Germany	61.1	80.7	14.3	52.7	18.8	12.8	1.4	8	18	23	46	73	19
Estonia	59.3	78.1	5.4	44.7	35.3	11.3	3.3	8	17	33	39	68	13
Lithuania	60.3	75.8	11.5	36.6	32.3	9.2	2.5	9	25	32	44	65	13
Latvia	54.2	74.5	6.1	30.7	38.4	16.6	6.4	9	25	32	31	61	17
Slovakia	57.3	77.0	10.1	46.1	28.4	6.7	1.5	6	21	40	37	66	19
Denmark	55.9	81.3	6.2	58.5	23.1	9.0	1.8	11	32	24	40	70	9
Sweden	66.5	83.1	6.3	63.0	23.8	6.1	0.8	9	31	23	31	65	8
Finland	57.9	81.2	7.3	66.7	19.4	5.4	0.8	18	27	32	37	71	14
Netherlands	58.5	81.7	10.6	65.1	19.4	3.4	0.9	7	43	24	51	67	7
Cluster A means	60.4	80.3	10.2	53.3	25.3	7.7	1.9	10.2	23.0	27.6	37.1	67.9	14.2
EU means	62.1	79.9	10.8	50.6	27.0	8.0	1.8	7.6	16.2	24.9	32.4	59.6	15.1
Croatia	60.3	77.7	5.8	39.7	39.5	9.9	2.1	6	11	28	31	52	23
Romania	59.0	75.1	5.4	39.8	37.7	11.8	1.6	2	5	34	35	59	23
Hungary	62.6	76.0	12.0	50.4	25.2	10.8	1.6	4	16	27	41	45	24
Italy	67.4	82.8	14.1	41.2	33.7	6.2	1.4	3	5	18	23	48	15
Portugal	59.1	81.8	19.0	46.9	24.2	7.0	2.1	4	4	22	21	49	21
Malta	70.2	82.4	5.5	56.4	28.2	6.2	1.5	7	4	15	17	48	11
Poland	62.4	77.2	15.5	58.4	18.3	5.7	0.7	2	6	20	28	41	23
Bulgaria	66.7	74.2	4.8	31.7	38.5	15.1	2.3	4	8	26	26	63	11
Czechia	61.8	79.0	4.4	38.6	39.8	10.6	1.4	7	12	19	21	65	11
Greece	67.0	80.8	17.3	40.7	27.7	10.7	3.5	4	8	21	28	54	5
Belgium	63.7	81.8	16.1	62.4	16.4	4.1	1.0	4	19	16	32	52	16
France	64.4	82.3	15.8	50.6	24.2	6.7	2.2	8	16	19	29	59	16
Cyprus	66.0	81.6	10.5	55.3	26.1	6.3	1.3	11	13	25	27	45	16
Austria	60.9	81.4	13.0	60.9	19.8	5.3	1.0	7	11	22	34	45	10
Cluster B means	63.7	79.6	11.4	48.1	28.5	8.3	1.7	5.2	9.9	22.3	28.1	51.8	16.1
EU means	62.1	79.9	10.8	50.6	27.0	8.0	1.8	7.6	16.2	24.9	32.4	59.6	15.1

Source: authors’ design using SPSS v.27.

## Data Availability

Data are available in a publicly accessible repository. The data presented in this study are openly available: https://europa.eu/eurobarometer/api/deliverable/download/file?deliverableId=83654 (accessed on 12 August 2024); https://ec.europa.eu/eurostat/databrowser/view/hlth_hlye__custom_12573060/default/table?lang=en (accessed on 12 August 2024); https://ec.europa.eu/eurostat/databrowser/view/ilc_pw08/default/table?lang=en (accessed on 12 August 2024).

## List of Acronyms

**Acronym**	**Definition**
PA	Physical activity
PI	Physical inactivity
HL	Healthy life
LE	Life expectancy
PH	Personal happiness
LTPA	Leisure-time physical activity
EU	European Union
HLYB	Healthy life years in absolute value at birth
LEB	Life expectancy in absolute value at birth
PH_A	Persons being happy in the last four weeks—Always
PH_MOTT	Persons being happy in the last four weeks—Most of the time
PH_S	Persons being happy in the last four weeks—Sometimes
PH_R	Persons being happy in the last four weeks—Rarely
PH_N	Persons being happy in the last four weeks—Never
PH_DK	Persons being happy in the last four weeks—I don’t know
EPS_R	Exercise or play sports—Regularly
EPS_WSR	Exercise or play sports—With some regularity
EPS_S	Exercise or play sports—Seldom
EPS_N	Exercise or play sports—Never
EPS_DK	Exercise or play sports—I don’t know
OPA_R	Other physical activity—Regularly
OPA_WSR	Other physical activity—With some regularity
OPA_S	Other physical activity—Seldom
OPA_N	Other physical activity—Never
OPA_DK	Other physical activity—I don’t know
VPR_1-3d	Vigorous physical activity—1–3 days
VPR_4-7d	Vigorous physical activity—4–7 days
VPR_N	Vigorous physical activity—Never
VPR_DK	Vigorous physical activity—I don’t know
MPR_1-3d	Moderate physical activity—1–3 days
MPR_4-7d	Moderate physical activity—4–7 days
MPR_N	Moderate physical activity—Never
MPR_DK	Moderate physical activity—I don’t know
W_1-3d	Walk for at least 10 min—1–3 days
W_4-7d	Walk for at least 10 min—4–7 days
W_N	Walk for at least 10 min—Never
W_DK	Walk for at least 10 min—I don’t know
S_L	Time spent sitting on a usual day—2 h 30 min or less
S_Mo	Time spent sitting on a usual day—2 h 31 min to 5 h 30 min
S_Mu	Time spent sitting on a usual day—5 h 31 min to 8 h 30 min
S_Vmu	Time spent sitting on a usual day—8 h 31 min or more
S_DK	Time spent sitting on a usual day—I don’t know

## References

[1] De Moor D. (2015). Walking Works: Making the Case to Encourage Greater Uptake of Walking as a Physical Activity and Recognise the Value and Benefits of Walking for Health.

[2] Hanson S., Jones A. (2015). Is there benefit that walking groups have health benefits? A systematic review and meta-analysis. Br. J. Sports Med..

[3] Eigenschenk B., Thomann A., McClure M., Davies L., Gregory M., Dettweiler U., Inglés E. (2019). Benefits of Outdoor Sports for Society. A Systematic Literature Review and Reflections on Evidence. Int. J. Environ. Res. Public Health.

[4] Blond K., Rasmussen M., Østergaard L., Gruntved A. (2016). Prospective Study of Bicycling and Risk of Coronary Heart Disease in Danish Men and Women. Circulation.

[5] Andre E.K., Williams N., Schwartz F., Bullard C. (2017). Benefits of Campus Outdoor Recreation Programs: A Review of the Literature. J. Outdoor Recreat. Educ. Leadersh..

[6] Marselle M.R., Irvine K.N., Warber S.L. (2014). Examining Group Walks in Nature and Multiple Aspects of Well–Being. Ecopsychology.

[7] Caspersen C.J., Powell K.E., Christenson G.M. (1985). Physical activity, exercise, and physical fitness: Definitions and distinctions for health-related research. Public Health Rep..

[8] Haseler T., Haseler C. (2022). Lack of physical activity is a global problem. BMJ.

[9] World Health Organization (2020). Guidelines on Physical Activity and Sedentary Behaviour: At a Glance.

[10] Prémusz V., Makai A., Ács P., Derkács E., Laczkó T. (2023). Association of Outdoor Physical Activity and Sports with Life Satisfaction among Women of Reproductive Age According to a European Representative Sample—A Longitudinal Analysis. Eur. J. Investig. Health Psychol. Educ..

[11] European Commission (2022). Special Eurobarometer 525 Report. https://europa.eu/eurobarometer/api/deliverable/download/file?deliverableId=83654.

[12] World Health Organization (2018). Global Action Plan on Physical Activity 2018–2030: More Active People for a Healthier World.

[13] Wren-Lewis S., Alexandrova A. (2021). Mental Health without Well–being. J. Med. Philos..

[14] Pilozzi A., Carro C., Huang X. (2021). Roles of ß–Endorphin in Stress, Behavior, Neuroinflammation, and Brain Energy Metabolism. Int. J. Mol. Sci..

[15] Marques A., Marconcin P., Werneck A.O., Ferrari G., Gouveia É.R., Kliegel M., Peralta M., Ihle A. (2021). Bidirectional Association between Physical Activity and Dopamine across Adulthood—A Systematic Review. Brain Sci..

[16] Eather N., Wade L., Pankowiak A., Eime R. (2023). The Impact of Sports Participation on Mental Health and Social Outcomes in Adults: A Systematic Review and the ‘Mental Health through Sport’ Conceptual Model. Syst. Rev..

[17] Wu J., Zhu L., Dong X., Sun Z., Cai K., Shi Y., Chen A. (2022). Relationship between Physical Activity and Emotional Regulation Strategies in Early Adulthood: Mediating Effects of Cortical Thickness. Brain Sci..

[18] Carreira Míguez M., Clemente Suárez V.J. (2023). Physical activity levels affect mental health and behavior in men. J. Men’s Health.

[19] Văduva C.C., Constantinescu C., Ţenovici M., Văduva A.R., Niculescu M., Dițescu D., Albu C.C., Albu D.F. (2016). Delayed interval delivery in twin pregnancy–Case reports. Rom. J. Morphol. Embryol..

[20] Semplonius T., Willoughby T. (2018). Long–Term Links between Physical Activity and Sleep Quality. Med. Sci. Sports Exerc..

[21] Kiecolt–Glaser J.K., Renna M.E., Shrout M.R., Madison A.A. (2020). Stress Reactivity: What Pushes Us Higher, Faster, and Longer—And Why It Matters. Curr. Dir. Psychol. Sci..

[22] Anderson E., Shivakumar G. (2013). Effects of Exercise and Physical Activity on Anxiety. Front. Psychiatry.

[23] Iwon K., Skibinska J., Jasielska D., Kalwarczyk S. (2021). Elevating Subjective Well–Being through Physical Exercises: An Intervention Study. Front. Psychol..

[24] Rosa D., Sabiston C.M., Kuzmocha-Wilks D., Cairney J., Darnell S.C. (2023). Group differences and associations among stress, emotional well–being, and physical activity in international and domestic university students. J. Am. Coll. Health.

[25] Reyes–Molina D., Alonso–Cabrera J., Nazar G., Parra–Rizo M.A., Zapata–Lamana R., Sanhueza–Campos C., Cigarroa I. (2022). Association between the Physical Activity Behavioral Profile and Sedentary Time with Subjective Well–Being and Mental Health in Chilean University Students during the COVID–19 Pandemic. Int. J. Environ. Res. Public Health.

[26] Schoeps K., de la Barrera U., Montoya–Castilla I. (2019). Impact of emotional development intervention program on subjective well–being of university students. High. Educ..

[27] Stubbs B.V., Smith L.D., Rosenbaum S., Schuch F., Firth J. (2018). Physical activity and mental health. Lancet Psychiatry.

[28] Buecker S., Simacek T., Ingwersen B., Terwiel S., Simonsmeier B.A. (2021). Physical activity and subjective well–being in healthy individuals: A meta-analytic review. Health Psychol. Rev..

[29] Brehm B.A., Iannotta J.G. (1998). Women and Physical Activity: Active Lifestyles Enhance Health and Well–Being. J. Health Educ..

[30] Warburton D.E., Nicol C.W., Bredin S.S. (2006). Health benefits of physical activity: The evidence. CMAJ.

[31] Montero M.P., Lopez–Gimenez M.R., Acevedo P., Mora A.I. (2015). Healthy aging: Gender and life-course perspective cycle. Eur. J. Investig. Health Psychol. Educ..

[32] Moreno–Murcia J.A., Belando N., Huáscar E., Torres M.D. (2017). Social support, physical exercise and life satisfaction in women. Rev. Latinoam. Psicol..

[33] Diener E. (1984). Subjective well–being. Psychol. Bull..

[34] Bondarev D., Sipilä S., Finni T., Kujala U.M., Aukee P., Kovanen V., Laakkonen E.K., Kokko K. (2021). Associations of physical performance and physical activity with mental well-being in middle-aged women. BMC Public Health.

[35] Schmiedeberg C., Schröder J. (2017). Leisure activities and life satisfaction: An analysis with German panel data. Appl. Res. Qual. Life.

[36] Martín–Talavera L., Gavín–Chocano Ó., Sanz–Junoy G., Molero D. (2023). Self–Concept and Self–Esteem, Determinants of Greater Life Satisfaction in Mountain and Climbing Technicians and Athletes. Eur. J. Investig. Health Psychol. Educ..

[37] Vega M.T. (2018). Sociocognitive model of life satisfaction in people with chronic disease. Eur. J. Investig. Health Psychol. Educ..

[38] Moore S.C., Lee I.-M., Weiderpass E., Campbell P.T., Sampson J.N., Kitahara C.M., Keadle S.C., Arem H., Berrington de Gonzalez A., Hartge P. (2016). Association of Leisure–Time Physical Activity with Risk of 26 Types of Cancer in 1.44 Million Adults. JAMA Intern. Med..

[39] Ding D., Lawson K.D., Kolbe–Alexander T.L., Finkelstein E.A., Katzmarzyk P.T., Van Mechelen W., Pratt M., Committee L.P.A.S.E. (2016). The economic burden of physical inactivity: A global analysis of major non-communicable diseases. Lancet.

[40] Albu D.F., Albu C.C., Văduva C.C., Niculescu M., Edu A. (2016). Diagnosis problems in a case of ovarian tumor—Case presentation. Rom J. Morphol. Embryol..

[41] Alvarez–Lourido D., Paniza Prados J.L., Alvarez–Sousa A. (2023). Ageing, Leisure Time Physical Activity and Health in Europe. Healthcare.

[42] Nikitara K., Odani S., Demenagas N., Rachiotis G., Symvoulakis E., Vardavas C. (2020). Prevalence and Correlates of Physical Inactivity in Adults across 28 European Countries. Eur. J. Public Health.

[43] Stalsberg R., Pedersen A.V. (2018). Are Differences in Physical Activity across Socioeconomic Groups Associated with Choice of Physical Activity Variables to Report?. Int. J. Environ. Res. Public Health.

[44] Thivel D., Tremblay A., Genin P.M., Panahi S., Rivière D., Duclos M. (2018). Physical Activity, Inactivity, and Sedentary Behaviors: Definitions and Implications in Occupational Health. Front. Public Health.

[45] Stalling I., Albrecht B.M., Foettinger L., Recke C., Bammann K. (2022). Associations between Socioeconomic Status and Physical Activity among Older Adults: Cross-Sectional Results from the OUTDOOR ACTIVE Study. BMC Geriatr..

[46] Eurostat Healthy Life Years by Sex. https://ec.europa.eu/eurostat/databrowser/view/hlth_hlye__custom_12573060/default/table?lang=en.

[47] Eurostat Persons Being Happy in the Last 4 Weeks by Sex, Age, Educational Attainment and Frequency. https://ec.europa.eu/eurostat/databrowser/view/ilc_pw08/default/table?lang=en.

[48] I.B.M (2012). SPSS—Neural Networks. https://www.ibm.com/downloads/cas/N7LLA2LB.

[49] Penn State, Eberly College of Science Agglomerative Hierarchical Clustering. https://online.stat.psu.edu/stat505/lesson/14/14.4.

[50] Hu Y., Li K., Meng A. Agglomerative Hierarchical Clustering using Ward Linkage. https://jbhender.github.io/Stats506/F18/GP/Group10.html.

[51] Shennar-Golan V.W.O. (2018). Physical Activity Intensity Among Adolescents and Association with Parent-Adolescent Relationship and Well–Being. Am. J. Men’s Health.

[52] Gualdi–Russo E., Zaccagni L. (2021). Physical Activity for Health and Wellness. Int. J. Environ. Res. Public Health.

[53] Belvederi Murri M., Folesani F., Zerbinati L., Nanni M.G., Ounalli H., Caruso R., Grassi L. (2020). Physical Activity Promotes Health and Reduces Cardiovascular Mortality in Depressed Populations: A Literature Overview. Int. J. Environ. Res. Public Health.

[54] Wang K., Wang X. (2020). Providing Sports Venues on Mainland China: Implications for Promoting Leisure–Time Physical Activity and National Fitness Policies. Int. J. Environ. Res. Public Health.

[55] White R.L., Babic M.J., Parker P.D., Lubans D.R., Astell-Burt T., Lonsdale C. (2017). Domain-Specific Physical Activity and Mental Health: A Meta-analysis. Am. J. Prev. Med..

[56] Rodrigues F., Morougo P., Santos T. (2023). Testing the Associations between Coping, Mental Health, and Satisfaction with Life in Portuguese Workers. Eur. J. Investig. Health Psychol. Educ..

[57] Puett R., Teas J., Espana–Romero V., Artero E., Duck-chul L., Baruth M., Sui X., Montresor–Lopez J., Blair S.N. (2014). Physical Activity: Does Environment Make a Difference for Tension, Stress, Emotional Outlook, and Perceptions of Health Status?. J. Phys. Act. Health.

[58] Fergusson D.M., McLeod G., Horwood L.J., Swain N.R., Chapple S., Poulton R. (2015). Life satisfaction and mental health problems (18 to 35 years). Psychol. Med..

[59] Mahindru A., Patil P., Agrawal V. (2023). Role of Physical Activity on Mental Health and Well–being: A Review. Cureus.

[60] Martín–Rodríguez A., Gostian–Ropotin L.A., Beltrán-Velasco A.I., Belando–Pedreño N., Simón J.A., López–Mora C., Navarro–Jiménez E., Tornero–Aguilera J.F., Clemente–Suárez V.J. (2024). Sporting Mind: The Interplay of Physical Activity and Psychological Health. Sports.

[61] Brown D.K., Barton J.L., Pretty J., Gladwell V.F. (2014). Walks4Work: Assessing the role of the natural environment in a workplace physical activity intervention. Scand. J. Work. Health.

[62] Clough P., Mackenzie S.H., Mallabon L., Brymer E. (2016). Adventurous physical activity environments: A mainstream intervention for mental health. Sports Med..

[63] Binsaeed R.H., Yousaf Z., Grigorescu A., Condrea E., Nassani A.A. (2023). Emotional Intelligence, Innovative Work Behavior, and Cultural Intelligence Reflection on Innovation Performance in the Healthcare Industry. Brain Sci..

[64] Akram U., Fülöp M.T., Tiron-Tudor A., Topor D.I., Căpușneanu S. (2021). Impact of Digitalization on Customers’ Well-Being in the Pandemic Period: Challenges and Opportunities for the Retail Industry. Int. J. Environ. Res. Public Health.

[65] Tong P., An I.S. (2024). Review of studies applying Bronfenbrenner’s bioecological theory in international and intercultural education research. Front. Psychol..

